# Prime Real Estate: Metals, Cofactors and MICOS

**DOI:** 10.3389/fcell.2022.892325

**Published:** 2022-05-20

**Authors:** Amy E. Medlock, J. Catrice Hixon, Tawhid Bhuiyan, Paul A. Cobine

**Affiliations:** ^1^ Department of Biochemistry and Molecular Biology, University of Georgia, Athens, GA, United States; ^2^ Augusta University/University of Georgia Medical Partnership, University of Georgia, Athens, GA, United States; ^3^ Department of Biological Sciences, Auburn University, Auburn, AL, United States

**Keywords:** metals, copper, iron, heme, MICOS complex

## Abstract

Metals are key elements for the survival and normal development of humans but can also be toxic to cells when mishandled. In fact, even mild disruption of metal homeostasis causes a wide array of disorders. Many of the metals essential to normal physiology are required in mitochondria for enzymatic activities and for the formation of essential cofactors. Copper is required as a cofactor in the terminal electron transport chain complex cytochrome c oxidase, iron is required for the for the formation of iron-sulfur (Fe-S) clusters and heme, manganese is required for the prevention of oxidative stress production, and these are only a few examples of the critical roles that mitochondrial metals play. Even though the targets of these metals are known, we are still identifying transporters, investigating the roles of known transporters, and defining regulators of the transport process. Mitochondria are dynamic organelles whose content, structure and localization within the cell vary in different tissues and organisms. Our knowledge of the impact that alterations in mitochondrial physiology have on metal content and utilization in these organelles is very limited. The rates of fission and fusion, the ultrastructure of the organelle, and rates of mitophagy can all affect metal homeostasis and cofactor assembly. This review will focus of the emerging areas of overlap between metal homeostasis, cofactor assembly and the mitochondrial contact site and cristae organizing system (MICOS) that mediates multiple aspects of mitochondrial physiology. Importantly the MICOS complexes may allow for localization and organization of complexes not only involved in cristae formation and contact between the inner and outer mitochondrial membranes but also acts as hub for metal-related proteins to work in concert in cofactor assembly and homeostasis.

## Introduction

**FIGURE 1:**
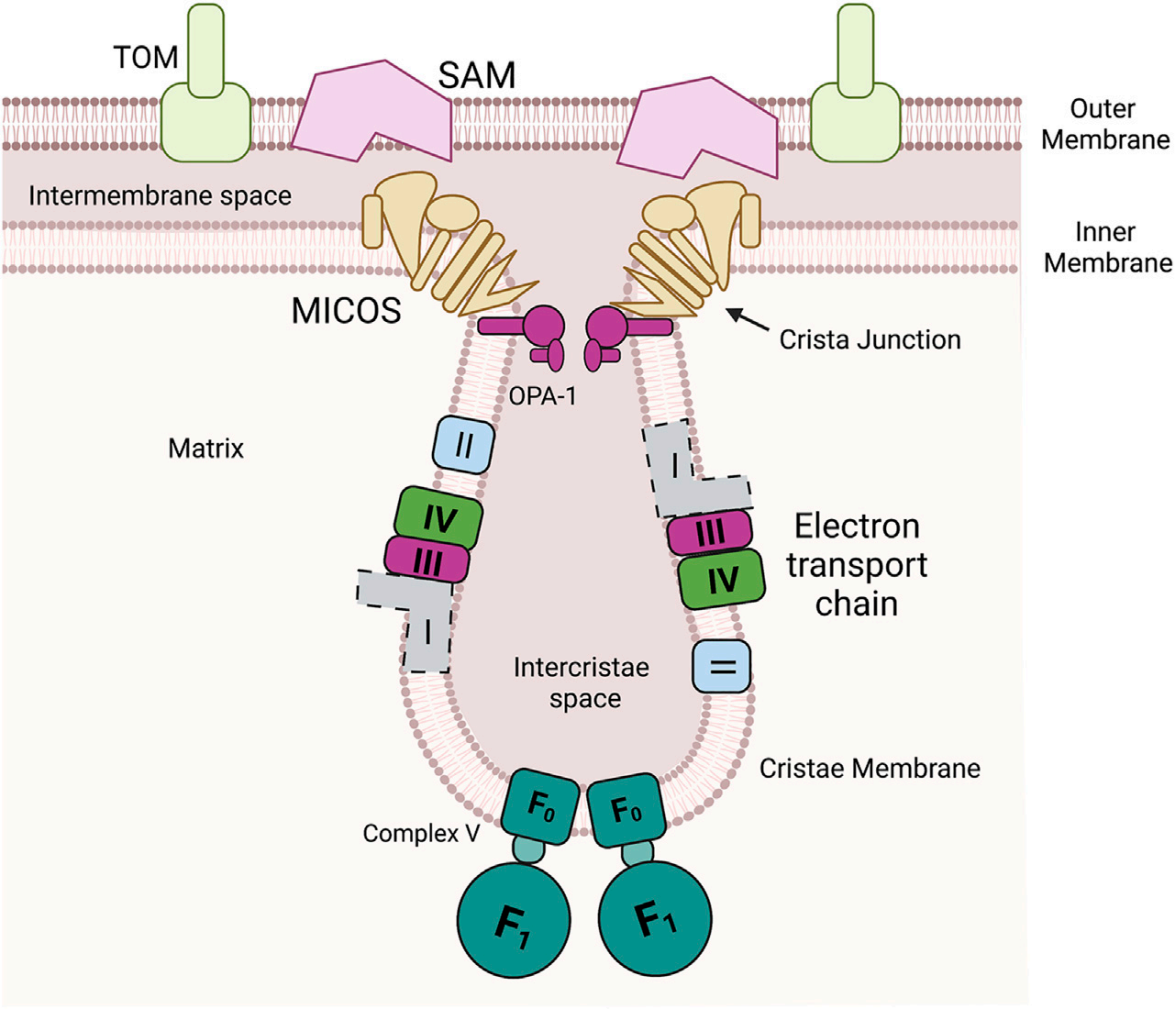
Mitochondria have multiple unique compartments. Outer membrane is a porous membrane that allows exchange of metabolites and small molecules with the cytosol but restricts the entry of proteins to the intermembrane space. The inner membrane is a highly protein loaded membrane that folds into invaginations known as cristae. The cristae house the assembled components of the oxidative phosphorylation (Complex I, II, III, and IV and ATP synthase (F_0_F_1_ ATPase, Complex V). In mammals this includes a membrane spanning NADH oxidoreductase (Complex I) (shown with dotted lines due differences between yeast and mammals), in yeast this enzyme is soluble in the matrix. The mitochondrial contact site and cristae organizing system (MICOS) and Opa-1 cooperate to form cristae junctions that encloses the intercristae space. MICOS also provides a structural point of contact between the inner and outer membrane via an interaction with the sorting and assembly machinery (SAM).

Mitochondria are a hub of metabolism and signaling (Cunningham and Rutter, 202020). At the heart of many of these activities are metal cofactors such as heme, iron sulfur clusters and other metalloenzymes (Pierrel et al., 2007a). Therefore, coordinating the flux of metals and controlling cofactor assembly should be closely linked with overall mitochondrial health. While the mitochondrial outer membrane (OM) is porous thus allowing for gated diffusion of metabolites, the inner membrane (IM) is folded into cristae and is impermeable to most small molecules and especially metals, Figure 1. Yet the internal matrix compartment requires metals for processing proteases as well as enzymes of the tricarboxcylic acid cycle. The IM houses many metalloenzymes and contains the highest ratio of proteins to lipids of all eukaryotic membranes (Kühlbrandt, 2015). The assembly and insertion of the cofactors into the complexes of electron transport chain requires assembly proteins localized in the intermembrane space (IMS) and the matrix (Vercellino and Sazanov, 2022). Some cofactors are produced (heme and Fe-S) or stored in the matrix (copper) and they must be redistributed to the IMS for assembly into the target enzymes (Cobine et al., 2021). Therefore, coordinating multiple proteins in multiple compartments with the availability of the metals/cofactors is a task that requires strategies to position the proteins involved at the right time in the right place.

The processes involved in mitochondrial ultrastructure, energetics, and metal homeostasis are conserved from yeast to mammals. While the nomenclature and naming of the proteins has been partially unified, examples of differently named homologs still exist. In this review we discuss a mixture of model systems that have been used to further our understanding of mitochondrial physiology. We have noted when evidence is from yeast or mammals in the text and have used all caps for protein names from mammals (including mouse and human) (e.g., SLC25A3) and have used sentence case (e.g., Pic2) for yeast proteins.

Multiple pathways evolved to facilitate the correct localization of the mitochondrial proteome (Dudek et al., 2013). Importantly, all the import pathways require proteins to be unfolded and therefore they must receive the cofactors required for activity in the organelle. Import and insertion is tightly regulated by protein complexes that allow transit of unfolded polypeptides to the correct location without causing disruption of the membrane potential as depolarization of mitochondria is a well described inducer of cell death. Using a combination of cytosolic and mitochondrial proteins, newly translated proteins enter the mitochondrion *via* translocase of the outer membrane (TOM). Then multiple pathways exist for the delivery of proteins to their final localization (Dudek et al., 2013). Two different TIM complexes, either Tim22-or Tim23-containing complexes, sort and facilitate the insertion of IM and matrix proteins, while the β-barrel sorting and assembly machinery (SAM) is required for OM protein insertion (Dudek et al., 2013). Cysteine-containing IMS localized proteins have a dedicated pathway called the mitochondrial intermembrane space import and assembly (MIA) machinery. Mia40 is the major protein that participates in import via transient disulfide bond formation with the incoming targets and then, in the final stages of the import process, facilitates the formation of disulfide bonds within the IMS protein (Dickson-Murray et al., 2021). In addition, the proteins known as the small TIMs assist in directing OM and IM proteins to the distinct complexes that facilitate insertion. The concerted action of all these complexes allows for the insertion and activity of the ∼1,000 nuclear encoded proteins that function in mitochondria.

Mitochondrial genomes content can vary in different organisms but generally it encodes ribosomal RNAs, tRNAs and select subunits of the OXPHOS machinery. The mRNAs encode the proteins that are core elements of the OXPHOS complexes (3 subunits of complex IV, 3 subunits of complex V, 1 subunit complex III in yeast; 3 subunits of complex IV, 2 subunits of complex V, 1 subunit complex III, 7 subunits of complex I in humans) are transcribed, translated within the matrix by a dedicated machinery. Once transcribed and translated these proteins are inserted into the IM to form distinct complexes and assembly intermediates that form in a modular fashion. OXA1 is essential for the correct insertion and assembly of many of these proteins (Hennon et al., 2015; Thompson et al., 2018). Mutations in human OXA1 result in fatal encephalitis, hypotonia, and developmental delay due to assembly defects in complexes I, IV and V (Thompson et al., 2018). Both human and yeast Oxa1 have been shown to interact with the mitochondrial ribosome. In yeast, mtDNA encoded subunits of complex V are synthesized on the cristae membrane, while complex III and IV components were synthesized both at the inner boundary membrane and cristae membrane as defined by the localization of the specific translational activators (Eaglesfield and Tokatlidis, 2021). Using fluorescent noncanonical amino acid tagging it was shown that in human cells the majority of mtDNA encoded proteins are synthesized at the cristae membrane (Zorkau et al., 2021). The authors of this study note that while the technique cannot follow synthesis in real time it is unlikely that the assembly intermediates could pass through the cristae junctions (Zorkau et al., 2021). Therefore, the cofactor containing subunits of OXPHOS requires the coordination of these translational factors, insertases, the available cofactor at specific localizations to ensure correct assembly.

## Mitochondrial Morphology and Organization

**FIGURE 2:**
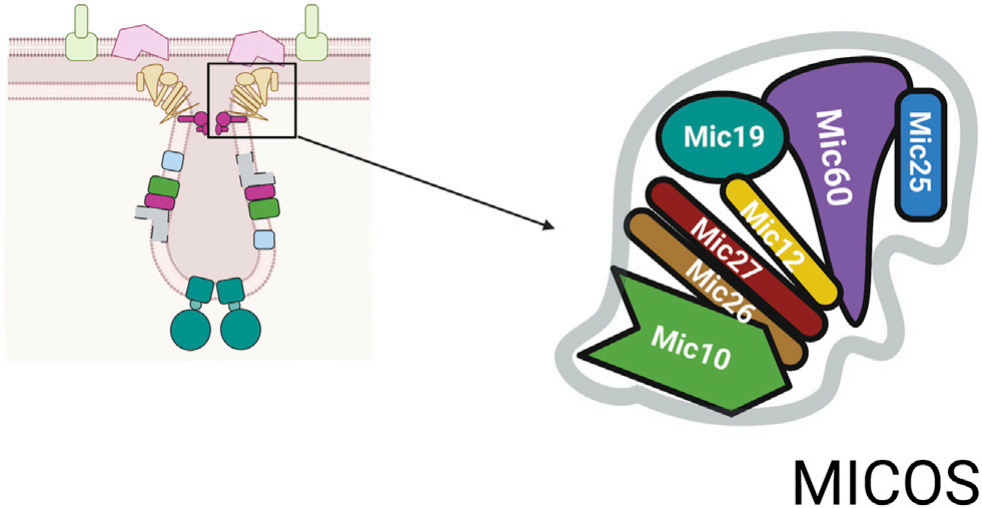
MICOS complex. The MICOS complex consists of multiple proteins named for their molecular weight. The numbers shown are based on mammalian system and are slightly different in yeast system.

Aberrant mitochondrial morphology is associated with cellular dysfunction suggesting that mitochondrial architecture and function are intimately linked. The mitochondrial contact site and cristae organizing system (MICOS) complex consists of numerous proteins essential for formation of mitochondrial structure, Figure 2 (Harner et al., 2011; Eramo et al., 2020). The MICOS complex establishes and maintains the inner membrane architecture, provides contacts between the IM and OM, and facilitates the closure of the cristae junction (Eramo et al., 2020). It is known that membrane potential, lipid and protein composition affect cristae formation and that the maintenance of cristae shape is linked to content of ATP synthase. The conserved dynamin related GTPase, OPA1 catalyzes membrane fusion and is found at crista junctions and regulates both the number of cristae and the release of cristae contents (Ehses et al., 2009).

Depending on the organism the number of core components identified to date varies (Eramo et al., 2020). The core components of MICOS are: Mic10, Mic12, Mic19, Mic25, Mic26, Mic27, and Mic60, Figure 2 (Pfanner et al., 2014). In addition, to the role in closing the cristae junction, the complex is required for the formation of contact sites between the IM and OM to facilitate communication between these two mitochondrial compartments. MICOS proteins also interact with proteins such as Ugo1, a part of mitochondrial fusion machinery, and porin, the abundant OM channel for small metabolites (Muñoz-Gómez et al., 2015). Through its links with multiple proteins, the MICOS complex functions as an organizer of mitochondrial architecture and integration platform for processes that are centered on mitochondrial membranes.

A recurring theme in mitochondria is that large complexes interact and work together exchanging proteins into and out the complexes to regulate functions critical to cellular physiology. This MICOS network includes interactions of Mic60 with the protein translocases TOM, SAM, and the oxidoreductase Mia40. Mic60 stimulates the TOM-SAM-mediated import of β-barrel proteins into the outer membrane forming a complex with TOM and SAM. In yeast, Tim23 and Mic60 interact to facilitate precursor handover from the TOM complex to the Tim23 complex by bringing the OM and IM into close contact. While TIM22 and MICOS work synergistically in human mitochondria to drive the translocation of mitochondria carrier family (MCF/SLC25) proteins (Callegari et al., 2019). Mic60 also interacts with the MIA machinery to mediate the import of intermembrane space proteins that have containing characteristic cysteine motifs (Rampelt et al., 2017). Since Mic60 transiently binds to both TOM and Mia40, Mic60 helps to position the receptor Mia40 to the intermembrane space side of the TOM complex. This spatial coupling function of Mic60 facilitates the formation of intermolecular disulfide bonds between Mia40 and the preprotein near the TOM complex preventing the exposure of potentially redox-sensitive cysteine residues in the preproteins. Thus, Mia40 can rapidly bind to precursor proteins passing through the TOM channel and promote their efficient import (Schorr and van der Laan, 2018). Mia40 pathway plays critical roles in the import of multiple metalloprotein and metalloprotein related assembly proteins. Therefore, MICOS has a critical role in the translocation of most mitochondrial proteins. This review will focus on the roles MICOS complexes have on metal homeostasis and the roles metalloproteins and metals have on MICOS stability.

## Transporters in Mitochondria

Membranes provide the greatest barrier to metal distribution in cells (Vest et al., 2013a). Therefore, understanding metal homoeostasis starts with understanding the transporters that facilitate entry and exit of the metals/cofactors. Transporters have the potential to act as scaffolds for loading metals however limitations in our ability to monitor these interactions, or a lack of knowledge about the transporters in particular compartments has slowed our progress in defining such roles. Mitochondria have five major classes of transporters in the IM that include the mitochondrial carrier family (MCF/SLC25), ATP-binding cassette (ABC) transporters, mitochondrial pyruvate carrier (MPC/SLC54), mitochondrial cation/H^+^ exchangers (LETM/SLC55), and sideroflexins (SLC56) (Cunningham and Rutter, 202020; Gyimesi and Hediger, 2020). Here we will focus on transporters involved in metals in mitochondria with the MPC and LETM families discussed elsewhere (Gyimesi and Hediger, 2020).

### Mitochondrial Carrier Family

Mitochondria contribute to intracellular signaling and regulatory networks in part by coordinating trafficking of metabolites into and out of mitochondria (Cunningham and Rutter, 202020). The mitochondrial carrier family (MCF/Slc25 family) form the largest group of transporters in the IM and are responsible for most of the metabolic flux to, and from, the organelle. In humans there are 53 members that are collectively responsible for the transport of numerous substrates including TCA cycle intermediates, nucleoside di- and triphosphates, amino acids, and metals (Cunningham and Rutter, 202020). Sixteen of the 53 MCFs have no known substrate and the established promiscuity of some of the characterized transporters raises the possibility that even those 37 with known substrates may have additional substrates. Notable members of this family involved in metals and metal cofactor assembly are SLC25A3/Pic2 which is a copper and phosphate transporter (Vest et al., 2013b; Boulet et al., 2018), SLC25A37/MITOFERRIN1/Mrs3 and SLC25A28/MITOFERRIN2/Mrs4 which are involved in mitochondrial iron homeostasis and necessary for iron transport (Shaw et al., 2006; Paradkar et al., 2009; Troadec et al., 2011; Christenson et al., 2018; Seguin et al., 2020), as well as SLC25A38 which transports glycine for heme synthesis (Guernsey et al., 2009).

MCF transporters have a conserved fold consisting of three repeats of approximately 100-amino acids that contain two transmembrane helices connected by a loop with a short α-helix (Ruprecht and Kunji, 2020). This structural insights into the family of transporters is based on the structure determined for ADP/ATP exchanger that was crystalized with inhibitors that stabilized the protein in either an open to the IMS state (c-state) or open to the matrix state (m-state) (Pebay-Peyroula et al., 2003; Ruprecht et al., 2019). The MCF fold is stabilized by salt bridges that form at the closed end of the substrate binding pocket depending on which state the protein adopts. The first,third, and fifth transmembrane helices contains a conserved PX (D/E)XX (R/K) motif that forms salt bridge and hydrogen bonding contacts on the matrix side of the binding pocket. A complementary (Y/F) (D/E) XX (R/K) motif in second,fourth, and sixth helices is found on the IMS side (Ruprecht and Kunji, 2020). The strength of these salt bridge interactions is an important predictor of the requirements and directionality of transport. Two examples exist of single amino-acids changes in MCF that give rise to changes in specificity. In yeast, Rim2 is a bifunctional pyrimidine and iron transporter but E248A mutant specifically disrupts mitochondrial iron transport activity while K299A mutant specifically abrogated pyrimidine nucleotide transport and exchange (Knight et al., 2019). Similarly, a L175A mutation can make the bifunctional copper and phosphate transporter SLC25A3 specific for copper transport (Zhu et al., 2021).

The metal transporting MCF SLC25A3 and SLC25A37 are predicted to be uniporters that import metals into the matrix. Both SLC25A3 and SLC25A37 have been successfully reconstituted into proteoliposomes to demonstrate transport activity (Boulet et al., 2018; Christenson et al., 2018). The yeast homologs, Pic2 and Mrs3 and mammalian SLC25A3 have also been expressed in *L. lactis.* Expression in *L. lactis* allows for monitoring import of metals into this organism. Both Pic2 and Mrs3 can transport copper in *L. lactis* although for Mrs3 to facilitate copper transport iron had to be chelated/limited presumably to prevent competition (Vest et al., 2016). The plant variant of SLC25A37 was also shown to have the ability to transport iron and copper (Bashir et al., 2011a; Bashir et al., 2011b). But our understanding about the interplay between these transporters *in vivo* is limited.

In addition to a role in transporting metals the MCF family has the potential to form a scaffold for protein-protein interactions to enhance the assembly of the mitochondrial metalloproteome. The MCF proteins have been isolated in many distinct large complexes. The large interactome of these proteins is exemplified by the ADP/ATP exchange proteins (Claypool et al., 2008; Claypool, 2009; Senoo et al., 2020). These proteins play critical role in delivery of the energy currency to the cell and have been isolated in large multiple protein complexes. These ADP/ATP exchanger protein complexes can also include other MCFs including the copper and phosphate carrier (SLC25A3), calcium dependent carriers (SLC25A13, SLC25A24, SLC25A25), the tricarboxylate carrier (SLC25A1), and the carnitine/acylcarnitine carrier (SLC25A20) (Claypool et al., 2008). How these interactions affect and/or regulate the specificity and/or transport activity is unknown due to the difficulties in assessing these biochemical characteristics *in vivo* or with purified components.

### ATP-Binding Cassette-Transporters

There are 4 mitochondrially-localized ABC-transporters (ABCB6, ABCB7, ABCB8 and ABCB10) which are part of a large superfamily of proteins that mediate nucleotide-dependent transport (Cunningham and Rutter, 202020). ABC-transporters have four core domains for function. Two transmembrane domains that provide specificity by forming a binding site, and two nucleotide binding domains to hydrolyze ATP to facilitate transport of the bound ligand. Conservation is found in the nucleotide binding domains with variability in the transmembrane domains to allow for a broad number of substrates (Wilkens, 2015). The four mitochondrial ABC-transporters are involved in moving Fe-S clusters and heme related metabolites into and out of mitochondria. ABCB6 is localized to the OM (and other cellular locations including the cell surface). ABCB6 has been proposed as porphyrin transporters (Krishnamurthy et al., 2006) and has been shown to specify the Langereis blood group (Helias et al., 2012).

**FIGURE 3:**
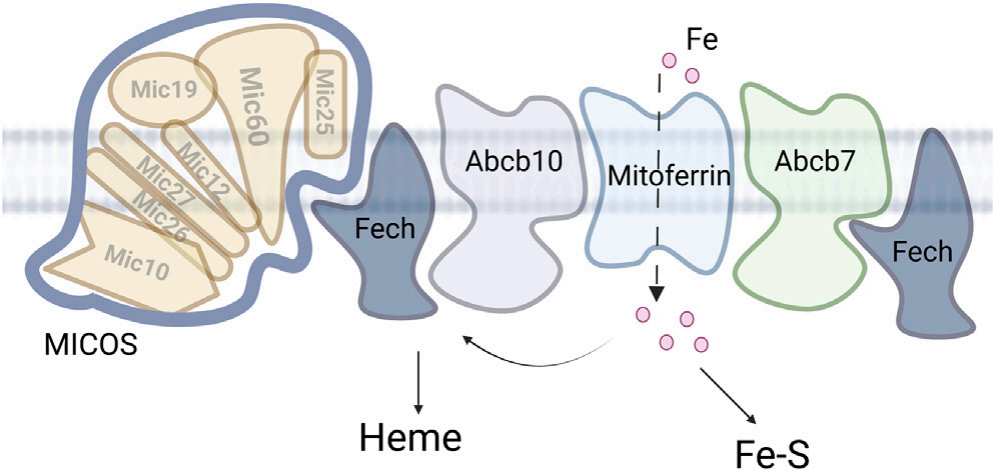
MICOS and mitochondrial iron. Iron enters the mitochondria *via* the MCF mitoferrin. The iron is then partitioned between heme synthesis, Fe-S assembly and other iron. Accumulation of the other iron is deleterious in multiple disease states.

ABCB7, ABCB8 and ABCB10 are all localized to the IM and the exact molecule transported by each is still unclear. ABCB7 links to cofactor assembly are because it was identified as a candidate causative gene for X-linked sideroblastic anemia with spinocerebellar ataxia and the yeast homolog *ATM1* is regulated by iron and participates in maintaining Fe-S cluster homeostasis (Pondarre et al., 2007; Lill, 2020). Similarly phenotypic data links ABCB8 to iron homeostasis as a mutation of *Abcb8* results in cardiomyopathy with increased mitochondrial iron accumulation with decreased activity of cytosolic Fe-S clusters proteins. Finally, the *Abcb10* knockout in mouse is embryonic lethal due to anemia. In support of a role of this transporter in iron homeostasis, *Abcb10* forms a complex with MITOFERRIN1 (SLC25A38) and ferrochelatase (FECH) that enhances heme synthesis, Figure 3 (Yamamoto et al., 2014; Medlock et al., 2015; Maio et al., 2019). While the substrate for ABCB10 has not be definitively identified, multiple studies suggest different substrates related to heme synthesis and it is clear this protein plays an important role in iron metabolism. The characterization of a larger complex that includes multiple heme synthesis related proteins is a continuation of the theme observed for the MCF proteins, that complexes of transporters and targets can form a potential regulatory mechanism for metal cofactor assembly or metal homeostasis.

### Other Transporters

Other notable mitochondrial transporters involved with metals are sideroflexins (Slc56). One of the five sideroflexin proteins, sideroflexin-1, has been shown to have a role in serine transport in mitochondria, and both sideroflexin-1 and sideroflexin-3 have been linked to iron homeostasis and Fe-S cluster biogenesis (Tifoun et al., 2021). Sideroflexins are 5 transmembrane domain proteins that contain a conserved mitochondrial tricarboxylate/iron carrier domain (PFAM: 03820). All members share the same topology with the amino-terminal inside and the carboxy-terminal outside. Sideroflexins 1–4 have sites of post-translational modification including acetylation that regulate activity and stability.

Cellular zinc transporters have been reported to localize to different mitochondrial membrane in some cell types. Zinc transporters can be broadly categorized in two groups ZIP (Irt-like) and ZnT (Kambe et al., 2014). ZIP transporters generally act as in zinc import while ZnT class act as zinc exporters. ZIP family members contain 8 transmembrane domains, with the proposed metal-binding residues embedded in transmembrane helices 4 and 5 and a cytoplasmic (inside/IMS in mitochondria) region between transmembrane 3 and transmembrane 4, that is, important for regulation including protein stability the exact roles of this domain are still under investigation (Kambe et al., 2014). It is also important to recognize that the ZIP family homologs have been shown to transport a variety of divalent ions including but not limited to iron, manganese, copper. Therefore, these transporters could have effects on other metals in mitochondria.

## Metals in Mitochondria

### Copper

Copper is found within each of the mitochondrial compartments. In the IM, cytochrome c oxidase is the multi-subunit complex that catalyzes the final steps of the electron transport chain (ETC). It accounts for about 25% of mitochondrial copper and is the major cuproenzyme present in this organelle (Cobine et al., 2004). In addition, a small percentage of cellular Cu, Zn superoxide dismutase (Sod1) and its copper chaperone, Ccs1, is localized to the IMS. However, these proteins do not account for a significant proportion of the total mitochondrial Cu. The majority (up to 70%) is found in the matrix and is bound to a biochemically characterized but structurally undefined complex known as the copper ligand (CuL) (Cobine et al., 2021). The copper in the matrix is transported by MCF SLC25A3/Pic2, that is, capable of transporting Cu and CuL as a substrate (Vest et al., 2013b; Boulet et al., 2018; Zhu et al., 2021). The data in multiple experimental models shows that the copper in the matrix is redistributed to the IMS for the assembly of cytochrome c oxidase (COX) and superoxide dismutase (Sod1). The matrix distribution model, that is, based on phenotypic data from mutants of the copper importer and biochemical competition assays in yeast under copper stress. These phenotypes are reproduced in mammalian cell culture without the need for copper restriction (Boulet et al., 2018). However, the identity of the transporter responsible for copper export is unknown and therefore the regulation of this pathway is poorly understood. An alternative pathway that feeds copper directly from the cytosol into these enzymes has been suggested based on purification of copper complexes that are thought to be in the IMS, this alternative pathway could operate under conditions where copper is abundant (Lindahl and Moore, 2016; Cobine et al., 2021).

Copper availability in the IMS is critical for the assembly of COX (Cobine et al., 2021). However, copper levels are restricted and therefore chaperone proteins are required to enhance the assembly process. This evidence for the copper delivery pathway is a combination of *in vitro* observations and genetic suppression experiments. In the IMS, Cox17 presents copper to Sco1 and Cox11 for assembly of the copper sites in COX called Cu_A_ and Cu_B_ sites, respectively (Horng et al., 2004; Cobine et al., 2021). Cox17 is a soluble protein that adopts a coiled coil-helix-coiled coil-helix fold stabilized by two disulfide bonds. The Cox17-Sco1 interaction is the most thoroughly studied copper transfer reaction that occurs in the IMS. Cox17 donates copper to the exposed CxxxC site on Sco1. Studies have shown that the reactions proceed from the Cu-loaded, partially oxidized conformer of human COX17 to SCO1 *via* transient interactions (Banci et al., 2007; Banci et al., 2008; Banci et al., 2011). This Cu-delivery pathway is further supported by the observation that *SCO1* overexpression can rescue the phenotype of a *COX17* mutant cell (Glerum et al., 1996). Sco1 subsequently insert the Cu into Cox2 to form the Cu_A_ site in COX. Multiple redox related steps are involved in the COX assembly process. Mammalian COA6 (and yeast Coa6) and SCO2 are two of the proteins with roles in regulating the reduction state of assembly proteins and the target COX2 (Pacheu-Grau et al., 2015; Soma et al., 2019; Swaminathan and Gohil, 2022). Most recently an additional interplay between the Cu assembly process and heme has been identified with a role discovered for COA7, a heme binding protein, that transiently interacts with the copper metallochaperones SCO1 and SCO2 and catalyzes the reduction of disulfide bonds within these proteins (Formosa et al., 2022). To build the Cu_B_ site, cells use a different battery of assembly proteins. Cox11 is a membrane bound factor required for the insertion of the Cu in COX1 and is also a recipient of copper from Cox17 (Baker et al., 2017a). Like Sco1 pathway specific accessory factors are required to maintain the redox state of the proteins in the pathway. Cox19 is required for the reduction of two critical copper binding cysteines in Cox11 (Bode et al., 2015). Copper transfer occurs *via* an interface than, that is, distinct from that used for interaction with Sco1 (Horng et al., 2004; Cobine et al., 2006). The requirement for this multi-step interplay of protein-protein interactions in the IMS to facilitate copper loading reinforces the idea that limited copper availability is a major complication for the correct metalation of cuproenzymes. It is also important to note that mutation in SCO proteins can cause remodeling of cellular copper homeostasis. The mutant cells and mice show copper deficiencies due to inappropriate degradation of the high affinity importer CTR1 or excess activity of the copper exporter ATP7A (Leary et al., 2007; Hlynialuk et al., 2015; Baker et al., 2017a; Baker et al., 2017b). During the cellular copper deficiency mitochondrial copper is maintained suggesting a possible prioritization of the matrix copper under these conditions (Dodani et al., 2011).

To date at least six gene deletions in yeast have been shown to limit matrix copper accumulation based on analysis of the purified mitochondria and mitochondrial targeted copper responsive reporters. The proteins implicated in copper import/maintenance are the MCF proteins: Pic2, Mrs3 and the assembly factors Coa1, Coa4, Coa6 and Shy1 (Pierrel et al., 2007b; Bestwick et al., 2010; Ghosh et al., 2014). Coa6 is a Cx_9_C containing protein of the IMS, that is, responsible for regulating the redox state of proteins involved in Cox2 assembly machinery (Ghosh et al., 2016; Soma et al., 2019; Swaminathan and Gohil, 2022). Coa1, Coa4 and Shy1 are all linked to the translation and assembly of Cox1 (Pierrel et al., 2007b). The mammalian homologs of Shy1 (SURF1) and Coa1 (COA1/MITRAC15) are part of the mitochondrial translation regulation assembly intermediate of cytochrome c oxidase (MITRAC) complex (Mick et al., 2012). This complex is another example of a large protein complexes that form in mitochondria to facilitate more efficient processing and/or substrate channeling. The assembly of the respiratory chain complexes proceeds in modular fashion and require complex-specific assembly factors to stabilize the intermediates for the complete maturation of complexes. Because maturation of the enzyme complexes is a sequential process during which new components and cofactors are added, the composition of the MITRAC complex changes during the process (Mick et al., 2012). Since MITRAC is facilitating the translation and assembly of COX1 and this is the heme and copper containing subunit it is intriguing to proposed that this complex could be critical for regulating total mitochondrial copper. It should be noted that only Coa1, Shy1 deletion and not other MITRAC homologs in yeast cause the copper deficiency.

### Iron

Iron enters mitochondria through multiple mechanisms. The most well-studied example of mitochondrial iron import is via MCF proteins MITOFERRIN1 in mammals and Mrs3 and Mrs4 in yeast (Dietz et al., 2021a). MITOFERRIN and Mrs3 have been shown to modulate the transport of iron *in vivo* and *in vitro* while Mrs4 is transcriptionally activated by iron depletion, suggesting a role for homeostasis of iron in mitochondria. Once iron crosses the IM it is used for the synthesis of heme, Fe-S or is bound by other ligands for storage. Genetic experiments suggested that FRAXATIN (or at least the yeast homolog Yfh1) played a role as a chaperone directing iron to produce Fe-S cluster largely based on an iron accumulation phenotype and the activation of Fe-regulated transcription in yeast in *YFH1* mutants (Llorens et al., 2019). However additional experimental evidence suggests that this is not a required function of Yfh1. The phenotypes of *yfh1∆* can be bypassed completely by a compensatory mutation in the Fe-S cluster scaffold protein IscU (Yoon et al., 2015). This combined with other approaches suggested the primary function of Yfh1 is sulfur delivery to form Fe-S clusters. Once iron reaches the iron-sulfur cluster machinery it is synthesized into 2Fe-2S clusters that are then inserted in mitochondrial targets or exported to the cytosol where the cytosolic iron-sulfur assembly (CIA) machinery matures them to 4Fe-4S clusters and/or inserts them into other targets (Lill, 2020). The other major iron cofactor is heme which is synthesized when iron is inserted into protoporphyrin IX by ferrochelatase (Dailey and Meissner, 2013). Protoporphyrin IX is synthesized by a series of enzymes that are split between the cytosol and the mitochondria with the initial steps of porphyrin synthesis taking place in mitochondria before export of the intermediates for maturation in the cytosol until the final steps occur back in the mitochondrion.

### Zinc and Manganese

The mechanisms of zinc and manganese uptake into the mitochondrial matrix are not well understood. Zinc is required in the mitochondria for multiple functions including electron transport chain function (COX), ATP synthesis (Atp32), protection against oxidative stress in the IMS (Sod1), lipid transport (Yme1L), mitochondrial dynamics (Oma1, Yme1L), processing peptidase (MPP), and mitochondrial intermediate peptidase (MIP). A genetic screen to identify a transporter using chemical sensors of available zinc uncovered an unexpected connection between Complex III assembly and mitochondrial zinc. A gene designated *MZM*1 was identified and characterized to show the gene product was required for both zinc maintenance and complex III assembly (Atkinson et al., 2010; Atkinson et al., 2011). However, this did not result in identification of a zinc transporter in mitochondria. Some studies have suggested that zinc can be imported to mitochondria by the calcium uniporter (MCU) (Gazaryan et al., 2007; Ji et al., 2020). While others have reported dual location of zinc transporters suggesting that zinc entry to the IMS could be facilitated by Zip1 that was shown to localize to the OM (Cho et al., 2019). Further still the zinc transporters Zip7 and ZnT7 have also been shown to have dual localization in mitochondria and ER and expression of Zip7 and ZnT7 contribute to cellular zinc exchange between the organelles in certain cell types (Tuncay et al., 2019). Finally Znt9 (SLC30A9) was identified as a zinc exporter in mitochondria (Deng et al., 2021) and SLC25A25 has been implicated in at least regulating uptake of zinc as deletion can reverse phenotypes induced by SLC30A9 deletion (Ma et al., 2022). Perhaps redundancy is the reason for the failure of the yeast genetic screens to yield a single candidate.

Manganese has a single target in the mitochondria in the form of superoxide dismutase 2 (SOD2). This matrix localized enzyme is responsible for protection against a subset of the reactive oxygen species generated. Deletion of *SOD2* in mammalian models is embryonic lethal demonstrating its essential role. However, to date no definitive data exists as the identity of the manganese transporter that provides this metal for the matrix for SOD2 assembly and activity. In yeast deletion of the gene *MTM1*, which encodes a MCF, caused decreased activity of Sod2 but did not prevent manganese accumulation (Luk et al., 2003). It was subsequently shown that deletion of *MTM1* changed iron availability resulting in mismetallation and inactivation of Sod2 by iron (Yang et al., 2006; Naranuntarat et al., 2009). Critically this is an example of the complex requirements for essential cofactor assembly in mitochondria and highlights the need for additional research on regulatory mechanisms that change availability.

## Cofactor Assembly

Mitochondria are essential for the synthesis of cofactors required for normal cellular function. It is widely accepted that synthesis of Fe-S clusters in mitochondria is the sole essential function for the organelle in eukaryotes. This is because divergent eukaryotes which have lost other mitochondrial function retain an organelle with Fe-S cluster assembly machinery. In addition to Fe-S clusters, mitochondria also house the machinery for assembly of heme and molybdenum cofactor (MoCo). Heme is required for all aerobic eukaryotes and is involved in oxygen metabolism, sterols synthesis, and amino acid biogenesis amongst other processes. MoCo is involved in multiple cellular functions including sulfur, drug, and nucleotide metabolism.

### Heme Assembly

Heme or Fe-protoporphyrin IX is an essential cofactor required for a plethora of cellular processes in eukaryotes. In metazoans, the heme biosynthesis is typically partitioned between the cytosol and mitochondria, with the first and final steps taking place in the mitochondrion. Key enzymes in the pathway that are found in the mitochondrial matrix are aminolevulinic acid synthase, which utilizes glycine and succinyl-CoA to produce aminolevulinic acid, and FECH, which utilizes iron and protoporphyrin IX to make heme (Phillips, 2019). Overall, the pathway has been well studied and all the biosynthetic enzymes structurally characterized. Nevertheless, understanding of the regulation of heme synthesis in different cells and factors that influence this process remains incomplete. Some details of the transcriptional regulation in the context of erythroid development are known, but transcriptional regulation in most other cell types or cellular conditions is much less understood (Dailey and Meissner, 2013). Recent work supports post-translational mechanisms for the regulation of the mitochondrial heme biosynthesis enzymes (Chung et al., 2017). Studies have shown protein-protein interactions regulate heme precursor levels (Medlock et al., 2015) and the incorporation or disruption of cofactors into key enzymes (Shah et al., 2012; Kardon et al., 2020).

### Iron-Sulfur Cofactor Assembly

Fe-S clusters are essential cofactors for the ETC and many other biochemical reactions and processes (Lill, 2020). The cofactor assembly systems are required to sequester, chaperone, and regulate delivery of the cofactor. The assembly proteins are not strictly required for building Fe-S cluster, because the clusters will self-assemble in solution, but is thought these proteins are necessary to circumvent the toxicity and indiscriminate reactivity of free iron and sulfide. Defects in the biosynthesis in Fe-S cluster cause dysfunction in mitochondria, neurodegenerative and cardiovascular disease, genomic instability, and the development of aging and cancer.

Mitochondria assemble not only mitochondrial Fe-S clusters but are also involved in the biosynthesis of Fe-S clusters for proteins in the cytosol and in the nucleus (Lill, 2020). These cytosolic and nuclear Fe-S cluster proteins with essential functions for example, ABC protein Rli1, which participates in ribosome assembly and ribosome recycling during termination of polypeptide synthesis (Kispal et al., 2005). Eukaryotic replicative DNA polymerases which also contain a Fe-S cluster in their C-terminal domain (Paul and Lill, 2015). The Fe-S cluster cofactor appears to be indispensable for efficient interaction with their accessory proteins during DNA replication.

### Molybdenum Cofactor

Molybdenum is a versatile redox element, that is, used by enzymes to catalyze diverse reactions. While not a focus of this review we will briefly discuss molybdenum cofactor (MoCo) as it is synthesized in mitochondria and has links to iron and copper. MoCo is an ancient cofactor involved in sulfur, drug, and nucleotide metabolism. MoCo synthesis begins in mitochondria with guanosine 5′-triphosphate which is converted *via* the two proteins to cyclic pyranopterin monophosphate (cPMP). Both mitochondrial enzymes in humans, MOCS1A and MOCS1B, are encoded by a single gene alternatively spliced to yield either protein. For MOCS1A exon one encodes the mitochondrial localization signal, and for MOCS1B localization is designated *via* exon 10 (Mayr et al., 2020). Interestingly MOCS1A can also be localized to the cytosol in primates via exon 1b, and while it appears to be functional its physiological role in the cytosol is unclear. MOCS1A belongs to the radical SAM enzyme superfamily and has two iron-sulfur clusters (Hänzelmann et al., 2004), specifically 4Fe-4S clusters, thus is connected to iron and iron-sulfur cluster homeostasis. cPMP is then exported across the mitochondria membranes to the cytosol where it is further modified (Mendel, 2013). In plants this is mediated by ATM3 which is the homolog of yeast Atm1 and mammalian ABCB7. Once in the cytosol MPT synthase transfers the two sulfurs to cPMP to create molydopterin (MPT). This intermediate can bind copper, and the copper may act as a protecting group as the cofactor synthesis proceeds (Mendel, 2013). The next step in synthesis is formation of the of adenylated-MPT before the final step of Mo insertion. Therefore, MoCo synthesis has overlap with copper and iron both utilizing cofactors and sharing a transport pathway.

## The Connection Between Metals and Organization

Mitochondria have a clear role in multiple aspects of metal homeostasis as described above. But many of the experimental approaches and techniques used have been “static” in nature and did not necessarily consider the dynamics of mitochondria. This includes consideration of total content, structure, and localization within the cell in different tissues and organisms. In a “two-way street” we have limited understanding of the impact that alterations in mitochondrial physiology have on metal content and utilization or how dynamic changes in mitochondria alter cofactor synthesis. Rates of fission and fusion, the ultrastructure of the organelle, and rates of mitophagy all have the possibility to impinge on metal homeostasis and cofactor assembly. An emerging area of overlap is between metal homeostasis/cofactor assembly and MICOS. As described previously the coordination of the proteins in larger complexes is a developing theme and we will discuss two interactions of MICOS with iron and copper proteins that regulate both cofactor assembly and mitochondrial function.

### Ferrochelatase-Mitochondrial Contact Site and Cristae Organizing System

The terminal enzyme of the heme biosynthesis pathway, ferrochelatase, has been identified to be part of a multi-protein complex or metabolon in the mitochondrial matrix. This complex has been studied in both mammalian cells (FECH) (Medlock et al., 2015) as well as yeast (Hem15) (Dietz et al., 2021b). In terms of the mammalian cells, most work has focused on the metabolon in developing erythroid cells, as these cells make a large amount of heme. An interesting finding was that mitochondrial heme metabolon interacted with MICOS proteins including MIC60, Figure 3 (Piel et al., 2016; Dietz et al., 2021b). Further work in yeast showed that loss of MICOS negatively impacts Hem15 activity, it affects the size of the Hem15 high-mass complex, and results in accumulation of reactive and potentially toxic porphyrins (which arise from the heme intermediates porphyrinogens) that may cause oxidative damage. Restoring intermembrane connectivity using a heterologously expressed protein capable of tethering the IM and OM “artificially” in MICOS-deficient cells mitigates these cytotoxic effects (Dietz et al., 2021b). The artificial tether clearly shows the importance of mitochondrial ultrastructure to the function of this enzyme.

The localization of ferrochelatase to MICOS also provides a connection to iron and Fe-S cluster trafficking at these sites. Ferrochelatase was shown to interact with SLC25A37/MITOFERRIN1/Mrs3, ABCB10, and ABCB7 (Taketani et al., 2003; Chen et al., 2010; Medlock et al., 2015; Maio et al., 2019)). The connection with MITOFERRIN and ABCB10 is clear as iron is a substrate for the enzyme and thus can be directly trafficked for heme synthesis. The role of FECH and ABCB7 (Banci et al., 2007; Banci et al., 2008) interaction is less clear, except that FECH itself has an 2Fe-2S cluster (Dailey et al., 1994) and thus may regulate or be regulated by Fe-S cluster flux in the matrix. Overall, these data provide new insights into how heme biosynthetic machinery is organized and regulated, linking mitochondrial architecture-organizing factors to heme synthesis for the efficient import of heme precursors and export of heme.

### COX17-Mitochondrial Contact Site and Cristae Organizing System

**FIGURE 4:**
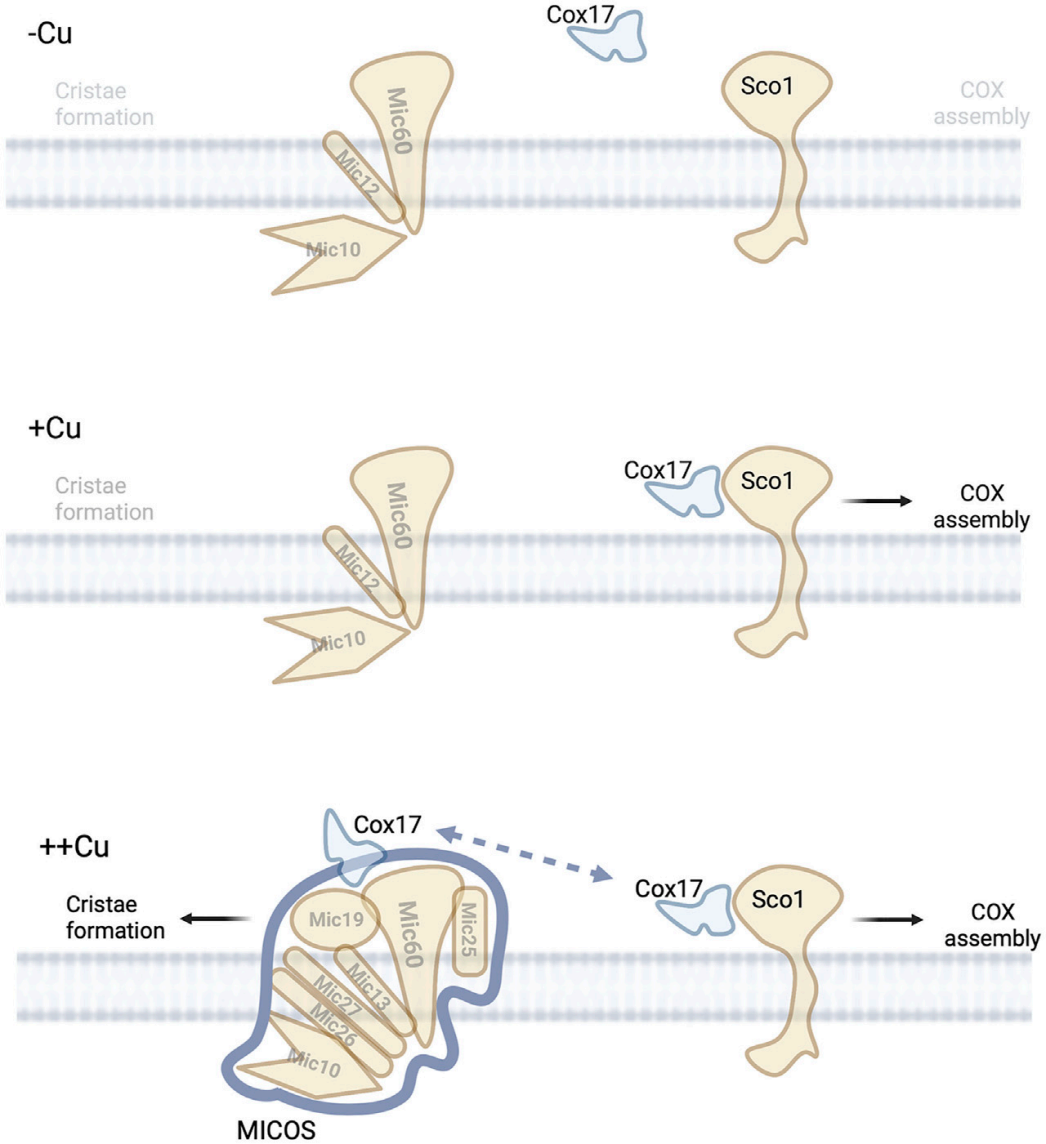
Copper and MICOS complex. Under low copper conditions no copper is available in the IMS to mediate the assembly of COX or allow for efficient assembly of MICOS suggesting low numbers of mitochondria cristae.

In addition to the role Cox17 plays in COX assembly, it also been linked to a copper dependent interaction with the MICOS complex (Vanisova et al., 2019; Chojnacka et al., 2015). Cox17 interacts with Mic60 to modulate MICOS complex integrity (Chojnacka et al., 2015). This interaction does not involve Sco1 or Cox11. However, the Cox17-MICOS interaction is regulated by copper, Figure 4. Cox17 is therefore a factor involved in maintaining the architecture of mitochondria *via* the MICOS complex. The regulation of the MICOS complex and therefore IM structure by Cox17 and copper was unexpected. However, it further consolidates a link between the mitochondrial morphology, ETC integrity, and metal homeostasis. It was proposed that Cox17 may be facilitating protein-protein interactions that enhance MICOS stability or that it could be delivering of copper to the MICOS complex (Chojnacka et al., 2015). This would require a yet to be identified copper-binding site in the complex. Cryptic sites of copper binding leading to metalloallosteric regulation have recently been described in multiple well studied processes (lipolysis, proliferation, and autophagy) and therefore a precedent exists to identify these new sites (Ge et al., 2022). Another possible model would be that the presence of excess Cu-Cox17 would indicate that COX is maximally metaled and therefore closing of cristae junctions should proceed to allow for enhanced activity of the OXPHOS in the enclosed intercristae space, Figure 4.

### SOD1-Mitochondrial Contact Site and Cristae Organizing System

The regulation of metal homeostasis is important to avoid the potential formation of reactive oxygen species (ROS). Superoxide dismutase (Sod1) is involved in the scavenging of ROS in the cytosol and in the IMS of mitochondria. Sod1 requires zinc and copper ions and an intramolecular disulfide bond to catalyzes the conversion of superoxide into hydrogen peroxide and water. The activation, localization, and retention (in IMS) of Sod1 is largely dependent upon the copper chaperone Ccs1 (Sturtz et al., 2001; Gross et al., 2011). Ccs1 is involved in the transfer of copper and disulfide bond formation in the maturation of Sod1. The assembly of Sod1 is also dependent on Mia40. Mia40 is responsible for import and oxidation of Ccs1 to the IMS. Ccs1 is responsible to “fold and trap” Sod1 in the IMS. An additional role for mitochondrial ultrastructure in Sod1 activity was uncovered. Mitochondria that are lacking any components of the MICOS complex can create unusual separations in the IMS. MICOS mutants accumulate a disproportionate amount of oxidized Sod1 (Varabyova et al., 2013). The authors speculate that the reduction of the disulfide bond in Sod1 is prevented by mitochondrial ultrastructure/compartmentalization (Varabyova et al., 2013). The metalation state of Sod1 in the MICOS mutants is unknown and perhaps mitochondrial copper status in MICOS mutations play some role in oxidation states of the Sod1. It is known that decreased mitochondrial copper can change the steady state levels of IMS localized Sod1.

Many mutations in human SOD1 are associated with amyotrophic lateral sclerosis (ALS), a nervous system disease that causes loss of muscle control. This debilitating disease has meant that numerous studies have investigated the role of mitochondrial SOD1 is disease progression. The accumulation of wildtype reduced Sod1 was dependent on the cristae architecture controlled by MICOS and mitochondria with MICOS defects showed an increase in mitochondrial accumulation of ALS-related reduced variants of Sod1 (Varabyova et al., 2013). This accumulation resulted in the accumulation of toxic superoxide and mitochondrial dysfunction (Varabyova et al., 2013). Further suggesting a role for correct mitochondrial ultrastructure in this disorder.

## Future Directions and Unanswered Questions

Until now our knowledge of metal homeostasis in mitochondria has been largely based on static measurements and the interpretation of genetic experiments. These have proven to be excellent resources for establishing the components of the pathways and identifying multiple interactors and suppressor that can bypass individual steps. However, recent technological advances in our ability to robustly detect protein complexes and our confidence that these interactions have bona fide physiological consequences have meant that we have learned that maintaining specific complexes in the highly crowded “real estate” of the mitochondrial IM is a recurring theme. The existence of interactions between copper and iron machinery and MICOS, that can serve as “a signpost to the most desirable neighborhood”, have triggered many additional questions about how mitochondrial morphology and metals are intertwined.

An outstanding question is what is the role of copper in the Cox17-MICOS interaction? Does this metal bind directly to stabilize a structure or perhaps enhance assembly or is the role of copper in stabilizing a fold of Cox17. The alpha-fold prediction of Mic60 does present multiple cysteine residues (as potential donors of thiol ligands) within 3–6 Å of each other (Jumper et al., 2021). The roles of these residues in metal binding has not been investigated. There have not been consistent reports of mitochondria morphology changes in cells lacking the major copper transporters such as CTR1, which restricts all available copper stores. However, this has not been systematically measured. In *SLC25A3* mutant cells, which have a localized copper deficiency in mitochondria, it has been reported that a less interconnected mitochondrial network and a mitochondrial fusion defect exists, that is, not explained by altered abundance of OPA1 or MITOFUSIN 1/2 or relative amount of different OPA1 forms (Seifert et al., 2016). This defect could be due to decreased mitochondrial copper which leads to changes in downstream targets such as COX17-MICOS complex. Additionally, yeast with lacking *MDM38,* a membrane-associated mitochondrial ribosome receptor with a role as K+/H+ exchange, have a mitochondrial fragmentation defect that can be suppressed by overexpression of *PIC2* and *MRS3* (Bauerschmitt et al., 2010; Zotova et al., 2010)*.* The mechanism of that rescue is linked to multiple physiological changes, but it is possible to speculate that *PIC2* and *MRS3* could be contributing to this rescue *via* copper transport function of these proteins. Further investigation would be required to pinpoint the mechanisms of defects and rescue of morphology in these mutants.

A major challenge for defining additional roles of the copper transporting MCF SLC25A3/Pic2 is the fact it has been identified in many complexes. In fact, SLC25A3 was included in the Contaminant Repository for Affinity Purification (CRAPome) due it is abundance and “stickiness” (Mellacheruvu et al., 2013). So, determining which complexes are “real” requires directed hypotheses. These hypotheses could include phenotypes such as the mitochondrial morphology under multiple conditions. While these experiments can be labor intensive, armed with the knowledge gained to date, it is now reasonable to invest the time. One observation that could be further investigated is SLC25A3 was found associated with MITRAC but not included as an interactor due to abundance in control samples (Mick et al., 2012). The possibility of SLC25A3 interaction to MITRAC complex would form an attractive link between COA1 and SURF1 (MITRAC members with mitochondrial copper defect) and copper. The mechanism of this is under investigation in yeast where *coa1∆* cells have decreased copper availability (Pierrel et al., 2007b). The copper deficit in mitochondria would limit the ability of Cox17 to complete its copper dependent interaction with MICOS raising the possibility of ultrastructure defect in this mutant. Unravelling this connection could further link MICOS to copper.

The interactions between MICOS and SAM and the affects that this could have on IMS content and connectivity to facilitate export of matrix components is understudied in metal homeostasis. The current model for copper and iron is that they are transported into the matrix by MCF proteins localized in the IM (Cobine et al., 2021). While transporters of iron in the OM have been proposed the directionality of the transport has not been completely resolved. For copper the model has always suggested that porins would act as IMS importers. If porin, or other proteins of the OM, were affected then the source of copper for SLC25A3/Pic2 could be disrupted. In addition, the model suggests that the copper chaperones (Cox17, Sco1, Cox11) are bypassed during import but used during export. Therefore, a link to mitochondrial ultrastructure and compartmentalization would help solve part of this bypass conundrum.

The assembly of heme and Fe-S are critical to aerobic eukaryotes and efforts to understand how the soluble metal is inserted into a hydrophobic molecule like protoporphyrin IX have long been discussed. The observation that this is mediated through a megacomplex answers some of the unresolved questions such as how do the substrates channel to the correct location and in part may also explain how a promiscuous enzyme such as ferrochelatase, that has been shown to insert multiple metals into the ring, can maintain specificity *in vivo*. Linking cofactor assembly to cristae morphology and therefore mitochondrial health is a way to coordinate investments of energy. If mitochondria are unable to produce heme or Fe-S then they can be eliminated as by mitophagy or at least reset by increased fission or fusion rates.

Clearly copper and iron are linked to mitochondrial ultrastructure changes. In addition, the uptake of zinc, manganese, and the export of the MoCo assembly intermediates could all depend on specific aspects of the MICOS machinery due to the fact that all must traverse the IMS during each process. Further understanding of the details of the metal and cofactor trafficking and the interactions with MICOS can provide information as to the regulation of these processes. It is possible that by locating these assembly proteins with a major complex that mediates critical morphology changes in the IM structure, means that metals and the cofactor assembly proteins have positioned themselves in an exclusive neighborhood and therefore are able to monitor, dictate and respond to changes in a dynamic manner to maintain cellular homeostasis and physiology.

## References

[B1] Atkinson A. Khalimonchuk O. Smith P. Sabic H. Eide D. Winge D. R. (2010). Mzm1 Influences a Labile Pool of Mitochondrial Zinc Important for Respiratory Function. J. Biol. Chem. 285, 19450–19459. 10.1074/jbc.m110.109793 20404342 PMC2885224

[B2] Atkinson A. Smith P. Fox J. L. Cui T.-Z. Khalimonchuk O. Winge D. R. (2011). The LYR Protein Mzm1 Functions in the Insertion of the Rieske Fe/S Protein in Yeast Mitochondria. Mol. Cell Biol. 31, 3988–3996. 10.1128/mcb.05673-11 21807901 PMC3187353

[B3] Baker Z. N. Cobine P. A. Leary S. C. (2017). The Mitochondrion: a Central Architect of Copper Homeostasis. Metallomics 9, 1501–1512. 10.1039/c7mt00221a 28952650 PMC5688007

[B4] Baker Z. N. Jett K. Boulet A. Hossain A. Cobine P. A. Kim B.-E. (2017). The Mitochondrial Metallochaperone SCO1 Maintains CTR1 at the Plasma Membrane to Preserve Copper Homeostasis in the Murine Heart. Hum. Mol. Genet. 26, 4617–4628. 10.1093/hmg/ddx344 28973536 PMC5886179

[B5] Banci L. Bertini I. Cefaro C. Ciofi-Baffoni S. Gallo A. (2011). Functional Role of Two Interhelical Disulfide Bonds in Human Cox17 Protein from a Structural Perspective. J. Biol. Chem. 286, 34382–34390. 10.1074/jbc.m111.246223 21816817 PMC3190761

[B6] Banci L. Bertini I. Ciofi-Baffoni S. Hadjiloi T. Martinelli M. Palumaa P. (2008). Mitochondrial Copper(I) Transfer from Cox17 to Sco1 Is Coupled to Electron Transfer. Proc. Natl. Acad. Sci. U.S.A. 105, 6803–6808. 10.1073/pnas.0800019105 18458339 PMC2383975

[B7] Banci L. Bertini I. Ciofi-Baffoni S. Leontari I. Martinelli M. Palumaa P. (2007). Human Sco1 Functional Studies and Pathological Implications of the P174L Mutant. Proc. Natl. Acad. Sci. U.S.A. 104, 15–20. 10.1073/pnas.0606189103 17182746 PMC1765425

[B8] Bashir K. Ishimaru Y. Nishizawa N. K. (2011). Identification and Characterization of the Major Mitochondrial Fe Transporter in Rice. Plant Signal. Behav. 6, 1591–1593. 10.4161/psb.6.10.17132 21921696 PMC3256392

[B9] Bashir K. Ishimaru Y. Shimo H. Nagasaka S. Fujimoto M. Takanashi H. (2011). The Rice Mitochondrial Iron Transporter Is Essential for Plant Growth. Nat. Commun. 2, 322. 10.1038/ncomms1326 21610725 PMC3113228

[B10] Bauerschmitt H. Mick D. U. Deckers M. Vollmer C. Funes S. Kehrein K. (2010). Ribosome-binding Proteins Mdm38 and Mba1 Display Overlapping Functions for Regulation of Mitochondrial Translation. MBoC 21, 1937–1944. 10.1091/mbc.e10-02-0101 20427570 PMC2883938

[B11] Bestwick M. Jeong M.-Y. Khalimonchuk O. Kim H. Winge D. R. (2010). Analysis of Leigh Syndrome Mutations in the Yeast SURF1 Homolog Reveals a New Member of the Cytochrome Oxidase Assembly Factor Family. Mol. Cell Biol. 30, 4480–4491. 10.1128/mcb.00228-10 20624914 PMC2937524

[B12] Bode M. Woellhaf M. W. Bohnert M. Laan M. v. d. Sommer F. Jung M. (2015). Redox-regulated Dynamic Interplay between Cox19 and the Copper-Binding Protein Cox11 in the Intermembrane Space of Mitochondria Facilitates Biogenesis of Cytochrome C Oxidase. MBoC 26, 2385–2401. 10.1091/mbc.e14-11-1526 25926683 PMC4571295

[B13] Boulet A. Vest K. E. Maynard M. K. Gammon M. G. Russell A. C. Mathews A. T. (2018). The Mammalian Phosphate Carrier SLC25A3 Is a Mitochondrial Copper Transporter Required for Cytochrome C Oxidase Biogenesis. J. Biol. Chem. 293, 1887–1896. 10.1074/jbc.ra117.000265 29237729 PMC5808751

[B14] Callegari S. Müller T. Schulz C. Lenz C. Jans D. C. Wissel M. (2019). A MICOS-TIM22 Association Promotes Carrier Import into Human Mitochondria. J. Mol. Biol. 431, 2835–2851. 10.1016/j.jmb.2019.05.015 31103774

[B15] Chen W. Dailey H. A. Paw B. H. (2010). Ferrochelatase Forms an Oligomeric Complex with Mitoferrin-1 and Abcb10 for Erythroid Heme Biosynthesis. Blood 116, 628–630. 10.1182/blood-2009-12-259614 20427704 PMC3324294

[B16] Cho H. M. Ryu J. R. Jo Y. Seo T. W. Choi Y. N. Kim J. H. (2019). Drp1-Zip1 Interaction Regulates Mitochondrial Quality Surveillance System. Mol. Cell 73, 364–376. 10.1016/j.molcel.2018.11.009 30581142

[B17] Chojnacka M. Gornicka A. Oeljeklaus S. Warscheid B. Chacinska A. (2015). Cox17 Protein Is an Auxiliary Factor Involved in the Control of the Mitochondrial Contact Site and Cristae Organizing System. J. Biol. Chem. 290, 15304–15312. 10.1074/jbc.m115.645069 25918166 PMC4463469

[B18] Christenson E. T. Gallegos A. S. Banerjee A. (2018). *In Vitro* reconstitution, Functional Dissection, and Mutational Analysis of Metal Ion Transport by Mitoferrin-1. J. Biol. Chem. 293, 3819–3828. 10.1074/jbc.m117.817478 29305420 PMC5846140

[B19] Chung J. Wittig J. G. Ghamari A. Maeda M. Dailey T. A. Bergonia H. (2017). Erythropoietin Signaling Regulates Heme Biosynthesis. Elife 6. 10.7554/eLife.24767 PMC5478267 28553927

[B20] Claypool S. M. (2009). Cardiolipin, a Critical Determinant of Mitochondrial Carrier Protein Assembly and Function. Biochimica Biophysica Acta (BBA) - Biomembr. 1788, 2059–2068. 10.1016/j.bbamem.2009.04.020 PMC2757529 19422785

[B21] Claypool S. M. Oktay Y. Boontheung P. Loo J. A. Koehler C. M. (2008). Cardiolipin Defines the Interactome of the Major ADP/ATP Carrier Protein of the Mitochondrial Inner Membrane. J. Cell Biol. 182, 937–950. 10.1083/jcb.200801152 18779372 PMC2528576

[B22] Cobine P. A. Moore S. A. Leary S. C. (2021). Getting Out what You Put in: Copper in Mitochondria and its Impacts on Human Disease. Biochimica Biophysica Acta (BBA) - Mol. Cell Res. 1868, 118867. 10.1016/j.bbamcr.2020.118867 PMC7680424 32979421

[B23] Cobine P. A. Ojeda L. D. Rigby K. M. Winge D. R. (2004). Yeast Contain a Non-proteinaceous Pool of Copper in the Mitochondrial Matrix. J. Biol. Chem. 279, 14447–14455. 10.1074/jbc.m312693200 14729672

[B24] Cobine P. A. Pierrel F. Leary S. C. Sasarman F. Horng Y.-C. Shoubridge E. A. (2006). The P174L Mutation in Human Sco1 Severely Compromises Cox17-dependent Metallation but Does Not Impair Copper Binding. J. Biol. Chem. 281, 12270–12276. 10.1074/jbc.m600496200 16520371

[B25] Cunningham C. N. Rutter J. (2020). 20,000 Picometers under the OMM: Diving into the Vastness of Mitochondrial Metabolite Transport. EMBO Rep. 21, e50071. 10.15252/embr.202050071 32329174 PMC7202207

[B26] Dailey H. A. Finnegan M. G. Johnson M. K. (1994). Human Ferrochelatase Is an Iron-Sulfur Protein. Biochemistry 33, 403–407. 10.1021/bi00168a003 8286370

[B27] Dailey H. A. Meissner P. N. (2013). Erythroid Heme Biosynthesis and its Disorders. Cold Spring Harb. Perspect. Med. 3, a011676. 10.1101/cshperspect.a011676 23471474 PMC3683999

[B28] Deng H. Qiao X. Xie T. Fu W. Li H. Zhao Y. (2021). SLC-30A9 Is Required for Zn(2+) Homeostasis, Zn(2+) Mobilization, and Mitochondrial Health. Proc. Natl. Acad. Sci. U. S. A. 118. 10.1073/pnas.2023909118 PMC8536367 34433664

[B29] Dickson-Murray E. Nedara K. Modjtahedi N. Tokatlidis K. (2021). The Mia40/CHCHD4 Oxidative Folding System: Redox Regulation and Signaling in the Mitochondrial Intermembrane Space. Antioxidants (Basel) 10. 10.3390/antiox10040592 PMC8069373 33921425

[B30] Dietz J. V. Fox J. L. Khalimonchuk O. (2021). Down the Iron Path: Mitochondrial Iron Homeostasis and beyond. Cells 10. 10.3390/cells10092198 PMC8468894 34571846

[B31] Dietz J. V. Willoughby M. M. Piel R. B. 3rd Ross T. A. Bohovych I. Addis H. G. (2021). Mitochondrial Contact Site and Cristae Organizing System (MICOS) Machinery Supports Heme Biosynthesis by Enabling Optimal Performance of Ferrochelatase. Redox Biol. 46, 102125. 10.1016/j.redox.2021.102125 34517185 PMC8441213

[B32] Dodani S. C. Leary S. C. Cobine P. A. Winge D. R. Chang C. J. (2011). A Targetable Fluorescent Sensor Reveals that Copper-Deficient SCO1 and SCO2 Patient Cells Prioritize Mitochondrial Copper Homeostasis. J. Am. Chem. Soc. 133, 8606–8616. 10.1021/ja2004158 21563821 PMC3106114

[B33] Dudek J. Rehling P. van der Laan M. (2013). Mitochondrial Protein Import: Common Principles and Physiological Networks. Biochimica Biophysica Acta (BBA) - Mol. Cell Res. 1833, 274–285. 10.1016/j.bbamcr.2012.05.028 22683763

[B34] Eaglesfield R. Tokatlidis K. (2021). Targeting and Insertion of Membrane Proteins in Mitochondria. Front. Cell Dev. Biol. 9, 803205. 10.3389/fcell.2021.803205 35004695 PMC8740019

[B35] Ehses S. Raschke I. Mancuso G. Bernacchia A. Geimer S. Tondera D. (2009). Regulation of OPA1 Processing and Mitochondrial Fusion by M-AAA Protease Isoenzymes and OMA1. J. Cell Biol. 187, 1023–1036. 10.1083/jcb.200906084 20038678 PMC2806285

[B36] Eramo M. J. Lisnyak V. Formosa L. E. Ryan M. T. (2020). The 'mitochondrial Contact Site and Cristae Organising System' (MICOS) in Health and Human Disease. J. Biochem. 167, 243–255. 10.1093/jb/mvz111 31825482

[B37] Formosa L. E. Maghool S. Sharpe A. J. Reljic B. Muellner-Wong L. Stroud D. A. (2022). Mitochondrial COA7 Is a Heme-Binding Protein with Disulfide Reductase Activity, Which Acts in the Early Stages of Complex IV Assembly. Proc. Natl. Acad. Sci. U. S. A. 119. 10.1073/pnas.2110357119 PMC8892353 35210360

[B38] Gazaryan I. G. Krasinskaya I. P. Kristal B. S. Brown A. M. (2007). Zinc Irreversibly Damages Major Enzymes of Energy Production and Antioxidant Defense Prior to Mitochondrial Permeability Transition. J. Biol. Chem. 282, 24373–24380. 10.1074/jbc.m611376200 17565998

[B39] Ge E. J. Bush A. I. Casini A. Cobine P. A. Cross J. R. DeNicola G. M. (2022). Connecting Copper and Cancer: from Transition Metal Signalling to Metalloplasia. Nat. Rev. Cancer 22, 102–113. 10.1038/s41568-021-00417-2 34764459 PMC8810673

[B40] Ghosh A. Pratt A. T. Soma S. Theriault S. G. Griffin A. T. Trivedi P. P. (2016). Mitochondrial Disease genesCOA6,COX6BandSCO2have Overlapping Roles in COX2 Biogenesis. Hum. Mol. Genet. 25, 660–671. 10.1093/hmg/ddv503 26669719 PMC4743686

[B41] Ghosh A. Trivedi P. P. Timbalia S. A. Griffin A. T. Rahn J. J. Chan S. S. L. (2014). Copper Supplementation Restores Cytochrome C Oxidase Assembly Defect in a Mitochondrial Disease Model of COA6 Deficiency. Hum. Mol. Genet. 23, 3596–3606. 10.1093/hmg/ddu069 24549041 PMC4049311

[B42] Glerum D. M. Shtanko A. Tzagoloff A. (1996). SCO1 and SCO2 Act as High Copy Suppressors of a Mitochondrial Copper Recruitment Defect in *Saccharomyces cerevisiae* . J. Biol. Chem. 271, 20531–20535. 10.1074/jbc.271.34.20531 8702795

[B43] Gross D. P. Burgard C. A. Reddehase S. Leitch J. M. Culotta V. C. Hell K. (2011). Mitochondrial Ccs1 Contains a Structural Disulfide Bond Crucial for the Import of This Unconventional Substrate by the Disulfide Relay System. Mol. Biol. Cell 22, 3758–3767. 10.1091/mbc.E11-04-0296 21865601 PMC3192856

[B44] Guernsey D. L. Jiang H. Campagna D. R. Evans S. C. Ferguson M. Kellogg M. D. (2009). Mutations in Mitochondrial Carrier Family Gene SLC25A38 Cause Nonsyndromic Autosomal Recessive Congenital Sideroblastic Anemia. Nat. Genet. 41, 651–653. 10.1038/ng.359 19412178

[B45] Gyimesi G. Hediger M. A. (2020). Sequence Features of Mitochondrial Transporter Protein Families. Biomolecules 10. 10.3390/biom10121611 PMC7761412 33260588

[B46] Hänzelmann P. Hernández H. L. Menzel C. García-Serres R. Huynh B. H. Johnson M. K. (2004). Characterization of MOCS1A, an Oxygen-Sensitive Iron-Sulfur Protein Involved in Human Molybdenum Cofactor Biosynthesis. J. Biol. Chem. 279, 34721–34732. 10.1074/jbc.m313398200 15180982

[B47] Harner M. Körner C. Walther D. Mokranjac D. Kaesmacher J. Welsch U. (2011). The Mitochondrial Contact Site Complex, a Determinant of Mitochondrial Architecture. EMBO J. 30, 4356–4370. 10.1038/emboj.2011.379 22009199 PMC3230385

[B48] Helias V. Saison C. Ballif B. A. Peyrard T. Takahashi J. Takahashi H. (2012). ABCB6 Is Dispensable for Erythropoiesis and Specifies the New Blood Group System Langereis. Nat. Genet. 44, 170–173. 10.1038/ng.1069 22246506 PMC3664204

[B49] Hennon S. W. Soman R. Zhu L. Dalbey R. E. (2015). YidC/Alb3/Oxa1 Family of Insertases. J. Biol. Chem. 290, 14866–14874. 10.1074/jbc.r115.638171 25947384 PMC4463434

[B50] Hlynialuk C. J. Ling B. Baker Z. N. Cobine P. A. Yu L. D. Boulet A. (2015). The Mitochondrial Metallochaperone SCO1 Is Required to Sustain Expression of the High-Affinity Copper Transporter CTR1 and Preserve Copper Homeostasis. Cell Rep. 10, 933–943. 10.1016/j.celrep.2015.01.019 25683716

[B51] Horng Y.-C. Cobine P. A. Maxfield A. B. Carr H. S. Winge D. R. (2004). Specific Copper Transfer from the Cox17 Metallochaperone to Both Sco1 and Cox11 in the Assembly of Yeast Cytochrome C Oxidase. J. Biol. Chem. 279, 35334–35340. 10.1074/jbc.m404747200 15199057

[B52] Ji S. G. Medvedeva Y. V. Weiss J. H. (2020). Zn2+ Entry through the Mitochondrial Calcium Uniporter Is a Critical Contributor to Mitochondrial Dysfunction and Neurodegeneration. Exp. Neurol. 325, 113161. 10.1016/j.expneurol.2019.113161 31881218 PMC6957126

[B53] Jumper J. Evans R. Pritzel A. Green T. Figurnov M. Ronneberger O. (2021). Highly Accurate Protein Structure Prediction with AlphaFold. Nature 596, 583–589. 10.1038/s41586-021-03819-2 34265844 PMC8371605

[B54] Kambe T. Hashimoto A. Fujimoto S. (2014). Current Understanding of ZIP and ZnT Zinc Transporters in Human Health and Diseases. Cell. Mol. Life Sci. 71, 3281–3295. 10.1007/s00018-014-1617-0 24710731 PMC11113243

[B55] Kardon J. R. Moroco J. A. Engen J. R. Baker T. A. (2020). Mitochondrial ClpX Activates an Essential Biosynthetic Enzyme through Partial Unfolding. Elife 9. 10.7554/eLife.54387 PMC7077987 32091391

[B56] Kispal G. Sipos K. Lange H. Fekete Z. Bedekovics T. Janáky T. (2005). Biogenesis of Cytosolic Ribosomes Requires the Essential Iron-Sulphur Protein Rli1p and Mitochondria. EMBO J. 24, 589–598. 10.1038/sj.emboj.7600541 15660134 PMC548650

[B57] Knight S. A. B. Yoon H. Pandey A. K. Pain J. Pain D. Dancis A. (2019). Splitting the Functions of Rim2, a Mitochondrial Iron/pyrimidine Carrier. Mitochondrion 47, 256–265. 10.1016/j.mito.2018.12.005 30660752 PMC6599733

[B58] Krishnamurthy P. C. Du G. Fukuda Y. Sun D. Sampath J. Mercer K. E. (2006). Identification of a Mammalian Mitochondrial Porphyrin Transporter. Nature 443, 586–589. 10.1038/nature05125 17006453

[B59] Kühlbrandt W. (2015). Structure and Function of Mitochondrial Membrane Protein Complexes. BMC Biol. 13, 89. 10.1186/s12915-015-0201-x 26515107 PMC4625866

[B60] Leary S. C. Cobine P. A. Kaufman B. A. Guercin G.-H. Mattman A. Palaty J. (2007). The Human Cytochrome C Oxidase Assembly Factors SCO1 and SCO2 Have Regulatory Roles in the Maintenance of Cellular Copper Homeostasis. Cell Metab. 5, 9–20. 10.1016/j.cmet.2006.12.001 17189203

[B61] Lill R. (2020). From the Discovery to Molecular Understanding of Cellular Iron-Sulfur Protein Biogenesis. Biol. Chem. 401, 855–876. 10.1515/hsz-2020-0117 32229650

[B62] Lindahl P. A. Moore M. J. (2016). Labile Low-Molecular-Mass Metal Complexes in Mitochondria: Trials and Tribulations of a Burgeoning Field. Biochemistry 55, 4140–4153. 10.1021/acs.biochem.6b00216 27433847 PMC5049694

[B63] Llorens J. V. Soriano S. Calap-Quintana P. Gonzalez-Cabo P. Moltó M. D. (2019). The Role of Iron in Friedreich's Ataxia: Insights from Studies in Human Tissues and Cellular and Animal Models. Front. Neurosci. 13, 75. 10.3389/fnins.2019.00075 30833885 PMC6387962

[B64] Luk E. Carroll M. Baker M. Culotta V. C. (2003). Manganese Activation of Superoxide Dismutase 2 in *Saccharomyces cerevisiae* Requires MTM1 , a Member of the Mitochondrial Carrier Family. Proc. Natl. Acad. Sci. U.S.A. 100, 10353–10357. 10.1073/pnas.1632471100 12890866 PMC193565

[B65] Ma T. Zhao L. Zhang J. Tang R. Wang X. Liu N. (2022). A Pair of Transporters Controls Mitochondrial Zn2+ Levels to Maintain Mitochondrial Homeostasis. Protein Cell 13, 180–202. 10.1007/s13238-021-00881-4 34687432 PMC8901913

[B66] Maio N. Kim K. S. Holmes-Hampton G. Singh A. Rouault T. A. (2019). Dimeric Ferrochelatase Bridges ABCB7 and ABCB10 Homodimers in an Architecturally Defined Molecular Complex Required for Heme Biosynthesis. Haematologica 104, 1756–1767. 10.3324/haematol.2018.214320 30765471 PMC6717564

[B67] Mayr S. J. Röper J. Schwarz G. (2020). Alternative Splicing of the Bicistronic Gene Molybdenum Cofactor Synthesis 1 (MOCS1) Uncovers a Novel Mitochondrial Protein Maturation Mechanism. J. Biol. Chem. 295, 3029–3039. 10.1074/jbc.ra119.010720 31996372 PMC7062190

[B68] Medlock A. E. Shiferaw M. T. Marcero J. R. Vashisht A. A. Wohlschlegel J. A. Phillips J. D. (2015). Identification of the Mitochondrial Heme Metabolism Complex. PLoS One 10, e0135896. 10.1371/journal.pone.0135896 26287972 PMC4545792

[B69] Mellacheruvu D. Wright Z. Couzens A. L. Lambert J.-P. St-Denis N. A. Li T. (2013). The CRAPome: a Contaminant Repository for Affinity Purification-Mass Spectrometry Data. Nat. Methods 10, 730–736. 10.1038/nmeth.2557 23921808 PMC3773500

[B70] Mendel R. R. (2013). The Molybdenum Cofactor. J. Biol. Chem. 288, 13165–13172. 10.1074/jbc.r113.455311 23539623 PMC3650355

[B71] Mick D. U. Dennerlein S. Wiese H. Reinhold R. Pacheu-Grau D. Lorenzi I. (2012). MITRAC Links Mitochondrial Protein Translocation to Respiratory-Chain Assembly and Translational Regulation. Cell 151, 1528–1541. 10.1016/j.cell.2012.11.053 23260140

[B72] Muñoz-Gómez S. A. Slamovits C. H. Dacks J. B. Wideman J. G. (2015). The Evolution of MICOS: Ancestral and Derived Functions and Interactions. Commun. Integr. Biol. 8, e1094593. 10.1080/19420889.2015.1094593 27065250 PMC4802753

[B73] Naranuntarat A. Jensen L. T. Pazicni S. Penner-Hahn J. E. Culotta V. C. (2009). The Interaction of Mitochondrial Iron with Manganese Superoxide Dismutase. J. Biol. Chem. 284, 22633–22640. 10.1074/jbc.m109.026773 19561359 PMC2755670

[B74] Pacheu-Grau D. Bareth B. Dudek J. Juris L. Vögtle F.-N. Wissel M. (2015). Cooperation between COA6 and SCO2 in COX2 Maturation during Cytochrome C Oxidase Assembly Links Two Mitochondrial Cardiomyopathies. Cell Metab. 21, 823–833. 10.1016/j.cmet.2015.04.012 25959673

[B75] Paradkar P. N. Zumbrennen K. B. Paw B. H. Ward D. M. Kaplan J. (2009). Regulation of Mitochondrial Iron Import through Differential Turnover of Mitoferrin 1 and Mitoferrin 2. Mol. Cell Biol. 29, 1007–1016. 10.1128/mcb.01685-08 19075006 PMC2643804

[B76] Paul V. D. Lill R. (2015). Biogenesis of Cytosolic and Nuclear Iron-Sulfur Proteins and Their Role in Genome Stability. Biochimica Biophysica Acta (BBA) - Mol. Cell Res. 1853, 1528–1539. 10.1016/j.bbamcr.2014.12.018 25583461

[B77] Pebay-Peyroula E. Dahout-Gonzalez C. Kahn R. Trézéguet V. Lauquin G. J.-M. Brandolin G. (2003). Structure of Mitochondrial ADP/ATP Carrier in Complex with Carboxyatractyloside. Nature 426, 39–44. 10.1038/nature02056 14603310

[B78] Pfanner N. van der Laan M. Amati P. Capaldi R. A. Caudy A. A. Chacinska A. (2014). Uniform Nomenclature for the Mitochondrial Contact Site and Cristae Organizing System. J. Cell Biol. 204, 1083–1086. 10.1083/jcb.201401006 24687277 PMC3971754

[B79] Phillips J. D. (2019). Heme Biosynthesis and the Porphyrias. Mol. Genet. Metabolism 128, 164–177. 10.1016/j.ymgme.2019.04.008 PMC7252266 31326287

[B80] Piel R. B. 3rd Shiferaw M. T. Vashisht A. A. Marcero J. R. Praissman J. L. Phillips J. D. (2016). A Novel Role for Progesterone Receptor Membrane Component 1 (PGRMC1): A Partner and Regulator of Ferrochelatase. Biochemistry 55, 5204–5217. 10.1021/acs.biochem.6b00756 27599036 PMC5278647

[B81] Pierrel F. Bestwick M. L. Cobine P. A. Khalimonchuk O. Cricco J. A. Winge D. R. (2007). Coa1 Links the Mss51 Post-translational Function to Cox1 Cofactor Insertion in Cytochrome C Oxidase Assembly. EMBO J. 26, 4335–4346. 10.1038/sj.emboj.7601861 17882260 PMC2034670

[B82] Pierrel F. Cobine P. A. Winge D. R. (2007). Metal Ion Availability in Mitochondria. Biometals 20, 675–682. 10.1007/s10534-006-9052-9 17225062

[B83] Pondarre C. Campagna D. R. Antiochos B. Sikorski L. Mulhern H. Fleming M. D. (2007). Abcb7, the Gene Responsible for X-Linked Sideroblastic Anemia with Ataxia, Is Essential for Hematopoiesis. Blood 109, 3567–3569. 10.1182/blood-2006-04-015768 17192398 PMC1852240

[B84] Rampelt H. Zerbes R. M. van der Laan M. Pfanner N. (2017). Role of the Mitochondrial Contact Site and Cristae Organizing System in Membrane Architecture and Dynamics. Biochimica Biophysica Acta (BBA) - Mol. Cell Res. 1864, 737–746. 10.1016/j.bbamcr.2016.05.020 27614134

[B85] Ruprecht J. J. King M. S. Zögg T. Aleksandrova A. A. Pardon E. Crichton P. G. (2019). The Molecular Mechanism of Transport by the Mitochondrial ADP/ATP Carrier. Cell 176, 435–447. 10.1016/j.cell.2018.11.025 30611538 PMC6349463

[B86] Ruprecht J. J. Kunji E. R. S. (2020). The SLC25 Mitochondrial Carrier Family: Structure and Mechanism. Trends Biochem. Sci. 45, 244–258. 10.1016/j.tibs.2019.11.001 31787485 PMC7611774

[B87] Schorr S. van der Laan M. (2018). Integrative Functions of the Mitochondrial Contact Site and Cristae Organizing System. Seminars Cell & Dev. Biol. 76, 191–200. 10.1016/j.semcdb.2017.09.021 28923515

[B88] Seguin A. Jia X. Earl A. M. Li L. Wallace J. Qiu A. (2020). The Mitochondrial Metal Transporters Mitoferrin1 and Mitoferrin2 Are Required for Liver Regeneration and Cell Proliferation in Mice. J. Biol. Chem. 295, 11002–11020. 10.1074/jbc.ra120.013229 32518166 PMC7415990

[B89] Seifert E. L. Gál A. Acoba M. G. Li Q. Anderson-Pullinger L. Golenár T. (2016). Natural and Induced Mitochondrial Phosphate Carrier Loss. J. Biol. Chem. 291, 26126–26137. 10.1074/jbc.m116.744714 27780865 PMC5207081

[B90] Senoo N. Kandasamy S. Ogunbona O. B. Baile M. G. Lu Y. Claypool S. M. (2020). Cardiolipin, Conformation, and Respiratory Complex-dependent Oligomerization of the Major Mitochondrial ADP/ATP Carrier in Yeast. Sci. Adv. 6, eabb0780. 10.1126/sciadv.abb0780 32923632 PMC7455186

[B91] Shah D. I. Takahashi-Makise N. Cooney J. D. Li L. Schultz I. J. Pierce E. L. (2012). Mitochondrial Atpif1 Regulates Haem Synthesis in Developing Erythroblasts. Nature 491, 608–612. 10.1038/nature11536 23135403 PMC3504625

[B92] Shaw G. C. Cope J. J. Li L. Corson K. Hersey C. Ackermann G. E. (2006). Mitoferrin Is Essential for Erythroid Iron Assimilation. Nature 440, 96–100. 10.1038/nature04512 16511496

[B93] Soma S. Morgada M. N. Naik M. T. Boulet A. Roesler A. A. Dziuba N. (2019). COA6 Is Structurally Tuned to Function as a Thiol-Disulfide Oxidoreductase in Copper Delivery to Mitochondrial Cytochrome C Oxidase. Cell Rep. 29, 4114–4126. 10.1016/j.celrep.2019.11.054 31851937 PMC6946597

[B94] Sturtz L. A. Diekert K. Jensen L. T. Lill R. Culotta V. C. (2001). A Fraction of Yeast Cu,Zn-Superoxide Dismutase and its Metallochaperone, CCS, Localize to the Intermembrane Space of Mitochondria. J. Biol. Chem. 276, 38084–38089. 10.1074/jbc.m105296200 11500508

[B95] Swaminathan A. B. Gohil V. M. (2022). The Role of COA6 in the Mitochondrial Copper Delivery Pathway to Cytochrome C Oxidase. Biomolecules 12. 10.3390/biom12010125 PMC8773535 35053273

[B96] Taketani S. Kakimoto K. Ueta H. Masaki R. Furukawa T. (2003). Involvement of ABC7 in the Biosynthesis of Heme in Erythroid Cells: Interaction of ABC7 with Ferrochelatase. Blood 101, 3274–3280. 10.1182/blood-2002-04-1212 12480705

[B97] Thompson K. Mai N. Oláhová M. Scialó F. Formosa L. E. Stroud D. A. (2018). OXA1L Mutations Cause Mitochondrial Encephalopathy and a Combined Oxidative Phosphorylation Defect. EMBO Mol. Med. 10. 10.15252/emmm.201809060 PMC6220311 30201738

[B98] Tifoun N. De Las Heras J. M. Guillaume A. Bouleau S. Mignotte B. Le Floch N. (2021). Insights into the Roles of the Sideroflexins/SLC56 Family in Iron Homeostasis and Iron-Sulfur Biogenesis. Biomedicines 9. 10.3390/biomedicines9020103 PMC7911444 33494450

[B99] Troadec M.-B. Warner D. Wallace J. Thomas K. Spangrude G. J. Phillips J. (2011). Targeted Deletion of the Mouse Mitoferrin1 Gene: from Anemia to Protoporphyria. Blood 117, 5494–5502. 10.1182/blood-2010-11-319483 21310927 PMC3109720

[B100] Tuncay E. Bitirim C. V. Olgar Y. Durak A. Rutter G. A. Turan B. (2019). Zn2+-transporters ZIP7 and ZnT7 Play Important Role in Progression of Cardiac Dysfunction via Affecting Sarco(endo)plasmic Reticulum-Mitochondria Coupling in Hyperglycemic Cardiomyocytes. Mitochondrion 44, 41–52. 10.1016/j.mito.2017.12.011 29307859

[B101] Vanisova M. Burska D. Krizova J. Danhelovska T. Dosoudilova Z. Zeman J. (2019). Stable COX17 Downregulation Leads to Alterations in Mitochondrial Ultrastructure, Decreased Copper Content and Impaired Cytochrome C Oxidase Biogenesis in HEK293 Cells. Folia Biol. (Praha) 65, 181–187. 31903891 10.14712/fb2019065040181

[B102] Varabyova A. Topf U. Kwiatkowska P. Wrobel L. Kaus-Drobek M. Chacinska A. (2013). Mia40 and MINOS Act in Parallel with Ccs1 in the Biogenesis of Mitochondrial Sod1. FEBS J. 280, 4943–4959. 10.1111/febs.12409 23802566

[B103] Vercellino I. Sazanov L. A. (2022). The Assembly, Regulation and Function of the Mitochondrial Respiratory Chain. Nat. Rev. Mol. Cell Biol. 23, 141–161. 10.1038/s41580-021-00415-0 34621061

[B104] Vest K. E. Hashemi H. F. Cobine P. A. (2013). The Copper Metallome in Eukaryotic Cells. Met. Ions Life Sci. 12, 451–478. 10.1007/978-94-007-5561-1_13 23595680

[B105] Vest K. E. Leary S. C. Winge D. R. Cobine P. A. (2013). Copper Import into the Mitochondrial Matrix in *Saccharomyces cerevisiae* Is Mediated by Pic2, a Mitochondrial Carrier Family Protein. J. Biol. Chem. 288, 23884–23892. 10.1074/jbc.m113.470674 23846699 PMC3745335

[B106] Vest K. E. Wang J. Gammon M. G. Maynard M. K. White O. L. Cobine J. A. (2016). Overlap of Copper and Iron Uptake Systems in Mitochondria in *Saccharomyces cerevisiae* . Open Biol. 6, 150223. 10.1098/rsob.150223 26763345 PMC4736827

[B107] Wilkens S. (2015). Structure and Mechanism of ABC Transporters. F1000Prime Rep. 7, 14. 10.12703/P7-14 25750732 PMC4338842

[B108] Yamamoto M. Arimura H. Fukushige T. Minami K. Nishizawa Y. Tanimoto A. (2014). Abcb10 Role in Heme Biosynthesis *In Vivo* : Abcb10 Knockout in Mice Causes Anemia with Protoporphyrin IX and Iron Accumulation. Mol. Cell Biol. 34, 1077–1084. 10.1128/mcb.00865-13 24421385 PMC3958026

[B109] Yang M. Cobine P. A. Molik S. Naranuntarat A. Lill R. Winge D. R. (2006). The Effects of Mitochondrial Iron Homeostasis on Cofactor Specificity of Superoxide Dismutase 2. EMBO J. 25, 1775–1783. 10.1038/sj.emboj.7601064 16601688 PMC1440838

[B110] Yoon H. Knight S. A. B. Pandey A. Pain J. Turkarslan S. Pain D. (2015). Turning *Saccharomyces cerevisiae* into a Frataxin-independent Organism. PLoS Genet. 11, e1005135. 10.1371/journal.pgen.1005135 25996596 PMC4440810

[B111] Zhu X. Boulet A. Buckley K. M. Phillips C. B. Gammon M. G. Oldfather L. E. (2021). Mitochondrial Copper and Phosphate Transporter Specificity Was Defined Early in the Evolution of Eukaryotes. Elife 10. 10.7554/elife.64690 PMC7924939 33591272

[B112] Zorkau M. Albus C. A. Berlinguer-Palmini R. Chrzanowska-Lightowlers Z. M. A. Lightowlers R. N. (2021). High-resolution Imaging Reveals Compartmentalization of Mitochondrial Protein Synthesis in Cultured Human Cells. Proc. Natl. Acad. Sci. U. S. A. 118. 10.1073/pnas.2008778118 PMC8017971 33526660

[B113] Zotova L. Aleschko M. Sponder G. Baumgartner R. Reipert S. Prinz M. (2010). Novel Components of an Active Mitochondrial K+/H+ Exchange. J. Biol. Chem. 285, 14399–14414. 10.1074/jbc.m109.059956 20197279 PMC2863244

